# Human liver‐expressed antimicrobial peptide 2 elevation in the cerebrospinal fluid in bacterial meningitis

**DOI:** 10.1002/brb3.2111

**Published:** 2021-04-03

**Authors:** Katsuya Sakai, Kazutaka Shiomi, Hitoshi Mochizuki, Md Nurul Islam, Hiroki Nabekura, Ryota Tanida, Hideyuki Sakoda, Masamitsu Nakazato

**Affiliations:** ^1^ Division of Neurology, Respirology, Endocrinology and Metabolism Department of Internal Medicine Faculty of Medicine University of Miyazaki Miyazaki Japan; ^2^ Department of Endocrinology and Metabolism Kanazawa University Graduate School of Medical Sciences Kanazawa Japan

**Keywords:** bacterial meningitis, cerebrospinal fluid, cross‐sectional study, enzyme‐linked immunosorbent assay, liver‐expressed antimicrobial peptide 2

## Abstract

**Objective:**

To study the presence of liver‐expressed antimicrobial peptide 2 (LEAP2) in human cerebrospinal fluid (CSF) and to measure its concentrations in neurological disorders.

**Materials & Methods:**

We identified the presence of LEAP2 in human CSF by chromatographic analysis and a LEAP2‐specific enzyme immunoassay. We measured LEAP2 concentrations in the CSF of 35 patients with neurological disorders.

**Results:**

CSF LEAP2 concentrations in the bacterial meningitis group (mean ± *SD*, 9.32 ± 3.76 ng/ml) were significantly higher (*p* < .05) than those in the other four groups (psychosomatic disorder, 0.56 ± 0.15 ng/ml; peripheral autoimmune disease, 1.00 ± 0.60 ng/ml; multiple sclerosis, 0.62 ± 0.30 ng/ml; aseptic meningitis, 1.59 ± 0.69 ng/ml).

**Conclusions:**

This is the first study to identify the presence of human LEAP2 in the CSF. Levels of LEAP2 were increased in the CSF of patients with bacterial meningitis. LEAP2 may have potential as a biomarker for bacterial meningitis.

## Graphical abstract

This original study describes the identification of liver‐expressed antimicrobial peptide 2 (LEAP2), an antimicrobial peptide, in human CSF, and is the first to measure CSF LEAP2 in neurological disorders. CSF LEAP2 is increased in patients with bacterial meningitis, suggesting its biological significance in the pathology of CNS infections.

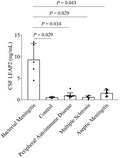

## 1 INTRODUCTION

Liver‐expressed antimicrobial peptide 2 (LEAP2) was first isolated from human hemofiltrate in 2003 as a cationic peptide consisting of 40 amino acids with two disulfide bonds (Krause et al., 2003). LEAP2 exhibited antimicrobial activity against the Gram‐positive bacteria *Bacillus megaterium, Bacillus subtilis, Micrococcus luteus,* and *Staphylococcus carnosus*; the Gram‐negative bacteria *Neisseria cinereal*; and the yeasts *Saccharomyces cerevisiae* and *Rhodotorula muciloginosa* (Krause et al., 2003). LEAP2 is named after its predominant expression in the mammalian liver. The effective antimicrobial concentration of LEAP2 (>6.6 µM = 30.2 µg/ml) has been shown to be more than 3,000‐fold higher than its plasma concentration in humans (~2 nM = 9.16 ng/ml) (Ge et al., 2018; Howard et al., 2010; Krause et al., 2003). Thus, for a long time its biological significance remained obscure, and LEAP2 was considered to act on the immune system rather than having a direct bactericidal effect.

In 2018, LEAP2 was shown to serve as an endogenous antagonist of the ghrelin receptor, known as growth hormone secretagogue receptor 1a (GHSR1a) (Ge et al., 2018). Ghrelin, a 28‐amino acid peptide, was originally discovered in the human and rat stomach as a hormone that stimulates food intake and growth hormone release (Kojima et al., 1999; Nakazato et al., 2001). A large number of studies have revealed that ghrelin also regulates the immune system by acting on immune cells and endothelial cells to suppress the production of pro‐inflammatory cytokines, suggesting its therapeutic viability in inflammatory conditions (Colldén et al., 2017; Yanagi et al., 2018). *Leap2* was found to be highly expressed in the liver and intestine in rodents, and its expression was decreased in the liver of rats under fasting condition (Islam et al., 2020). LEAP2 was also demonstrated to act as an inverse agonist of GHSR1a by blocking its constitutive activity (M’Kadmi et al., 2019). LEAP2 suppressed food intake and growth hormone release and maintained viable glucose levels during chronic caloric restriction in mice (Ge et al., 2018). The six N‐terminal amino acids of LEAP2 are crucial for GHSR1a binding, and its 10‐amino‐acid N‐terminal fragment stimulated insulin release from human pancreatic islets (Hagemann et al., 2020; Wang et al., 2019). Plasma LEAP2 concentrations in humans increased with body weight gain and elevations of blood glucose and decreased with fasting and weight loss surgery (Mani et al., 2019).

Although GHSR1a is widely expressed in the central nervous system (CNS) (Al‐Massadi et al., 2018), it is still unclear whether LEAP2 is involved in human neurological diseases. We demonstrated that the intracerebroventricular administration of LEAP2 to rats antagonized central ghrelin functions, including the promotion of food intake, elevation of blood glucose levels, and reduction of body temperature (Islam et al., 2020). Previous studies have not shown whether LEAP2 is present in human cerebrospinal fluid (CSF), and if so, whether its levels are altered in neurological disorders.

## 2 MATERIALS AND METHODS

### 2.1 Study population

This study enrolled 35 patients, specifically five with bacterial meningitis (four with *Streptococcus pneumoniae* and one with *Listeria monocytogenes*), five with viral meningitis, four with cryptococcal meningitis, five with chronic inflammatory demyelinating polyneuropathy (CIDP), five with Guillain–Barré syndrome (GBS), five with multiple sclerosis (MS), one with *Enterococcus faecalis* bacteremia but no CNS infection, and five with psychosomatic disorder as controls. These patients were admitted to our hospital between April 2014 and March 2020, and CSF was examined immediately after admission. Patients with CIDP, GBS, and MS were diagnosed according to European Federation of Neurological Societies/Peripheral Nerve Society clinical criteria (Van den Bergh et al., 2010), diagnostic criteria proposed by a Dutch consensus group in 2001 (Van der Meche et al., 2001), and the McDonald diagnostic criteria (Polman et al., 2011), respectively. In the patient with bacteremia, *Enterococcus faecalis* was detected by blood culture, while her CSF profile demonstrated a negative Gram stain, normal cell count, and normal protein and glucose concentrations. In addition, cytology and culture results were within normal limits. The patients were classified into five categories (Table 1): bacterial meningitis (Pneumococcal meningitis and Listeria meningitis), peripheral autoimmune disease (GBS and CIDP), multiple sclerosis, aseptic meningitis (cryptococcal meningitis and viral meningitis), and controls (psychosomatic disorder). The patient with bacteremia without CNS infection was excluded from these categories. We also measured fasting serum LEAP2 levels in 12 healthy individuals (age: 38.4 ± 9.2 years, eight men and four women) who had no notable medical history or underlying disease. The study protocol was approved by the Ethics Committee of the University of Miyazaki, with a waiver of written informed consent from patients and healthy volunteers, and was carried out according to the Declaration of Helsinki.

**TABLE 1:** Characteristics of the study groups

Disease type	Control group	Peripheral autoimmune disease		Central autoimmune disease	Aseptic meningitis		Bacterial meningitis	
	Psychosomatic disorder	Guillain–Barré syndrome	Chronic inflammatory demyelinating polyneuropathy	Multiple sclerosis	Viral meningitis	Cryptococcal meningitis	Listeria meningitis	Pneumococcal meningitis
Number	5	5	5	5	5	4	1	4
Sex								
Women	3	3	3	5	3	0	1	3
Men	2	2	2	0	2	4	0	1
Age	33.8 ± 16.2	49.6 ± 18.7	67.8 ± 9.9	42.6 ± 6.3	57.0 ± 10.1	69.0 ± 7.2	65	60.3 ± 20.2

### 2.2 Chromatographic characterization of CSF LEAP2

The N‐terminus of LEAP2 is a methionine residue whose thiol molecule is susceptible to oxidization in the normal atmosphere. To explore the presence of oxidized LEAP2 in human CSF, we oxidized synthetic LEAP2 (Peptide Institute) by the hydrogen peroxide method (Williams et al., 2010). Synthetic human LEAP2 (1 nmol) was reacted in a solution containing 1 N formic acid and 0.06% hydrogen peroxide for 30 min at 30°C. This peptide was analyzed by reverse‐phase high‐performance liquid chromatography (RP‐HPLC) (Chromaster; Hitachi) using a TSKgel ODS‐120T column (Tosoh Corporation) with a linear gradient of 10%–60% acetonitrile containing 0.1% trifluoroacetic acid (TFA) for 40 min at a flow rate of 1 ml/min. The absorbance of the eluent was monitored at 210 nm. To identify LEAP2 in human CSF, the CSF obtained by lumbar puncture in a patient with idiopathic normal pressure hydrocephalus was first treated in a 10‐kDa column (AmiconUltra 0.5 ml Centrifugation Filters; Merck Millipore) to collect substances whose molecular masses were less than 10 kDa. Next, the substances were analyzed by RP‐HPLC under the conditions described above. HPLC fractions separated every 30 s were analyzed with an enzyme‐linked immunosorbent assay (ELISA; LEAP2 (38–77) (Human)/LEAP2 (37–76) (Mouse))–enzyme immunoassay (EIA) Kit (Phoenix Pharmaceuticals). This ELISA detected oxidized and nonoxidized LEAP2 with equal sensitivity on a molar basis.

### 2.3 Measurements of LEAP2 in CSF and serum

Frozen 100 µl serum samples kept at − 80°C were allowed to melt at room temperature. Samples were loaded onto a Sep‐Pak Vac C18 cartridge (Waters Corporation) prepared as described elsewhere (Mitsukawa et al., 1990). Substances were eluted with 60% acetonitrile containing 0.1% TFA, and then the eluate was lyophilized. Serum LEAP2 concentrations were measured with the ELISA kit. CSF samples were collected by lumbar puncture, immediately centrifuged, and stored at − 80°C. CSF samples (100 µl) were centrifuged at 100 × *g* for 5 min to exclude the cell components, and then 50‐µl supernatant aliquots were measured with the ELISA kit. The correlation between CSF storage periods and CSF LEAP2 concentrations was analyzed.

### 2.4 Data analysis

Data were analyzed by one‐way ANOVA with the post hoc Games‐Howell test for intergroup analysis. The correlations between CSF LEAP2 and body mass index (BMI), serum glucose, C‐reactive protein (CRP), CSF protein, and CSF cell count were analyzed by Spearman's correlation coefficient test. Data are expressed as means ± *SD*, and significance was assumed at *p* < .05. IBM SPSS version 25 was used for statistical tests.

## 3 RESULTS

### 3.1 Identification of LEAP2 in human CSF

**FIGURE 1:**
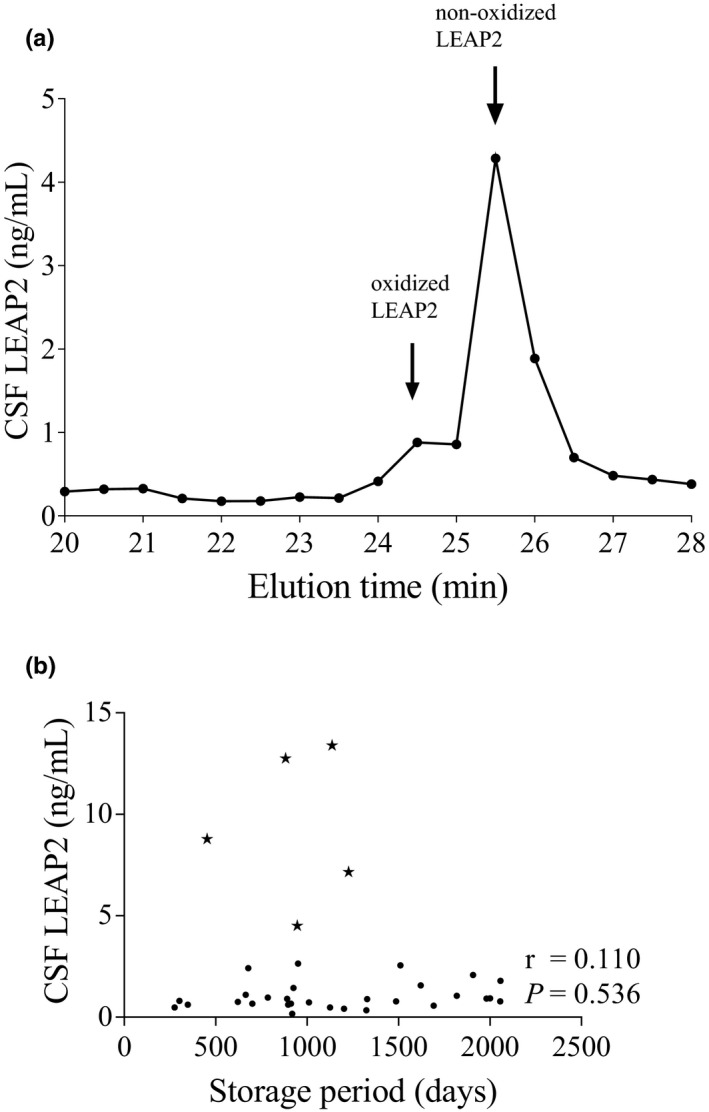
(a) Chromatographic characterization of LEAP2 in 18 ml of human CSF from a patient with idiopathic normal pressure hydrocephalus. Arrows indicate the elution positions of oxidized and nonoxidized LEAP2 according to elution time. (b) Correlation between CSF LEAP2 concentrations and the CSF storage period. Asterisks represent the data of patients with bacterial meningitis. Solid circles show the data of the control group and patients with peripheral autoimmune disease, multiple sclerosis, and aseptic meningitis. Data were analyzed by Spearman's correlation test

To identify the presence of LEAP2 in human CSF, we analyzed LEAP2‐immunoreactive substances in the CSF by RP‐HPLC. ELISA analysis of human CSF samples showed two immunoreactive peaks (Figure 1a). Their elution positions were identical to those of oxidized and nonoxidized LEAP2. The molar ratio of oxidized LEAP2 to nonoxidized LEAP2 was 1:4.9. CSF LEAP2 concentrations were not affected by the CSF storage period, which ranged from 32 days to 2,058 days (Figure 1b).

### 3.2 CSF LEAP2 concentrations in bacterial meningitis

**FIGURE 2:**
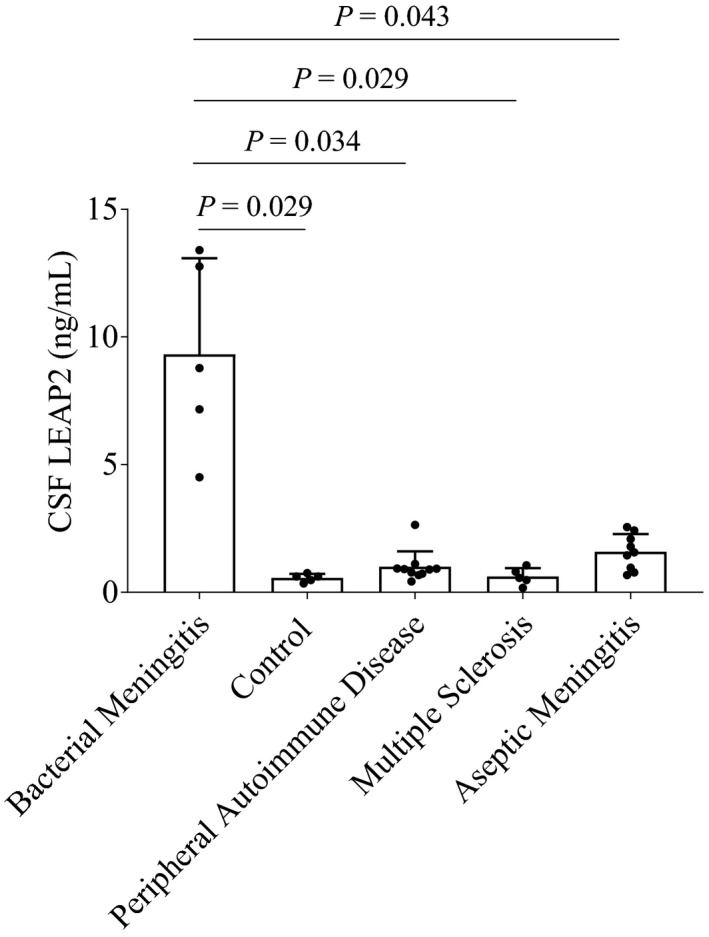
Increased LEAP2 concentrations in the CSF of bacterial meningitis. Comparative analysis of CSF LEAP2 concentrations among the five groups. Data were analyzed by one‐way ANOVA followed by the Games‐Howell test

**FIGURE 3:**
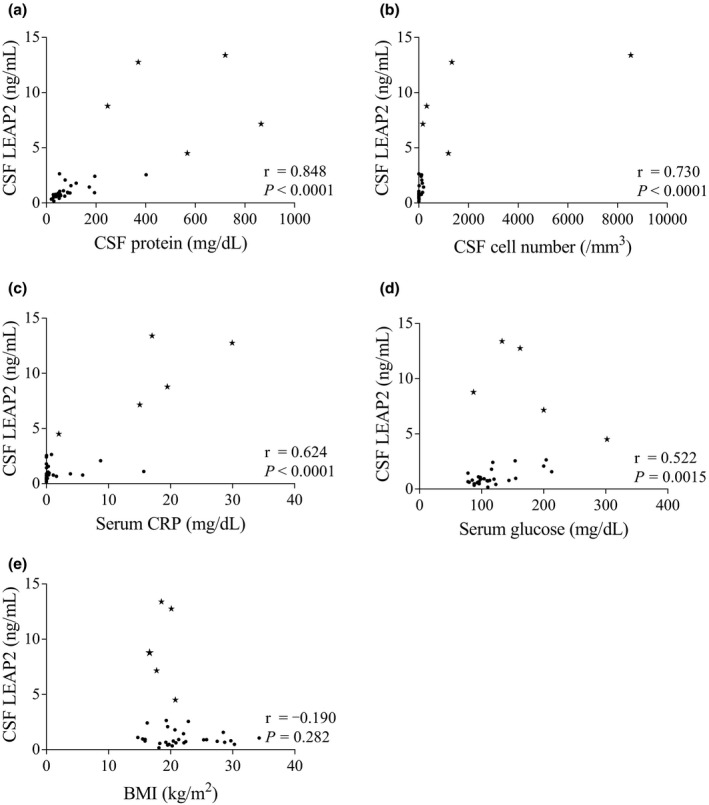
Correlation between CSF LEAP2 concentrations and inflammatory or metabolic parameters. Relationship between CSF LEAP2 concentrations and (a) CSF protein concentration, (b) CSF cell count, (c) serum CRP, (d) serum glucose, and (e) BMI. Asterisks show the data of patients with bacterial meningitis. Solid circles show the data of the control group and patients with peripheral autoimmune disease, multiple sclerosis, and aseptic meningitis. Data were analyzed by Spearman's correlation test

CSF LEAP2 concentrations in the bacterial meningitis group were significantly higher than those in the other four clinical groups (Figure 2). LEAP2 concentrations in the CSF of the patients in all five groups were positively correlated with CSF protein concentration, cell count, and serum CRP concentration (Figure 3a–c). CSF LEAP2 correlated with serum glucose, but not with BMI (Figure 3d, e). The CSF LEAP2 concentration of the Listeria meningitis patient decreased from 7.16 ng/ml to 3.04 ng/ml after antibiotic treatment.

### 3.3 Serum LEAP2 in bacterial infection

The serum LEAP2 concentrations in the 12 healthy subjects ranged from 6.89 ng/ml to 28.84 ng/ml (mean ± *SD*, 18.35 ± 7.15 ng/ml) (Table 2). The serum LEAP2 concentration in the patient with Listeria meningitis (13.47 ng/ml) was also within this range, while that in the patient with bacteremia (31.40 ng/ml), who had no CNS infection, was higher than the range observed in the healthy subjects. The CSF LEAP2 concentration was lower in the bacteremia patient (2.53 ng/ml) than in the five patients with bacterial meningitis (ranging from 4.51 ng/ml to 13.40 ng/ml; mean ± *SD*, 9.32 ± 3.76 ng/ml).

**TABLE 2:** LEAP2 concentrations in CSF and serum

	Bacterial meningitis		Bacteremia	Volunteer
	Listeria	Pneumococcus		
Age	65	60.3 ± 26.0	69	38.4 ± 9.2
Sex	W	W 3/M 1	W	W 4/M 8
CSF LEAP2 (ng/mL)				
Pretreatment	7.16	9.86 ± 4.11	2.53	not available
Posttreatment	3.04	not available	not available	not available
Serum LEAP2 (ng/mL)				
Pretreatment	13.47	not available	31.40	18.35 ± 7.15

Note

Abbreviations: M, men; W, women.

## 4 DISCUSSION

In this study, we first verified the presence of LEAP2 in human CSF by RP‐HPLC combined with ELISA. LEAP2 has an N‐terminal methionine whose thiomethyl residue is readily oxidized under conventional conditions (Davies, 2016). We hypothesized that a portion of LEAP2 might be oxidized in the CSF. We demonstrated that both methionine‐oxidized and nonoxidized LEAP2 were present in the CSF.

LEAP2 penetrates the negatively charged bacterial membrane because of its characteristic strong basicity and two disulfide bonds, causing holes or pores that eventually lead to bacterial cell death. This behavior affects the membranes of bacteria more preferentially than those of eukaryotes (Townes et al., 2009). Serum LEAP2 in mice was shown to antagonize ghrelin action on its cognate receptor and also to suppress ghrelin production (Ge et al., 2018). By contrast, ghrelin suppressed LEAP2 expression in the liver via the ghrelin receptor (Islam et al., 2020). Ghrelin exerts a broad spectrum of physiological actions, including modulating immunity and inflammation and reducing oxidative stress (Colldén et al., 2017; Yanagi et al., 2018). Considering LEAP2’s antagonism of ghrelin, LEAP2 may also be involved in modulating the immune system. Although plasma LEAP2 concentrations in patients with rheumatoid arthritis were reported to be higher than those in healthy subjects (Francisco et al., 2020), the role of LEAP2 in inflammation has not yet been clarified.

In this study, we showed that patients with bacterial meningitis had significantly higher CSF LEAP2 concentrations than those with other neurological disorders and a control group. CSF LEAP2 concentrations positively correlated with inflammatory parameters in the CSF and serum. The CSF LEAP2 concentration in the patient with Listeria meningitis declined during the recovery phase. Elevated CSF LEAP2 concentrations appear to reflect bacterial infection in the CNS.

The patient with bacteremia had a normal CSF LEAP2 concentration but an elevated serum LEAP2 concentration, while the reverse was true in the patient with Listeria meningitis. Another antimicrobial peptide, LL‐37, was produced in astrocytes and microglial cells in a rat model of pneumococcal meningitis (Brandenburg et al., 2008). Ghrelin can cross the blood–CSF barrier in mice (Uriarte et al., 2019). GHSR1a is highly expressed in various areas of the mouse brain, including the choroid plexus, hippocampus, hypothalamic arcuate nucleus, paraventricular nucleus, median eminence, area postrema, nucleus of the solitary tract, and ventral tegmental area (Perello et al., 2019). LEAP2 mRNA is expressed in the rat brain, including in the cerebral cortex, hypothalamus, hippocampus, olfactory bulb, cerebellum, midbrain, and medulla oblongata, although at low levels (Islam et al., 2020). Although it is unclear whether LEAP2 enters the brain by the same mechanism as ghrelin, LEAP2 levels rise in the human CNS during bacterial infection.

Inflammation produces a variety of anorectic substances that reduce food intake (Roxburgh and McMillan, 2014). Intraventricular administration of LEAP2 suppressed ghrelin‐induced feeding in rats (Islam et al., 2020). The elevated level of CSF LEAP2 in bacterial meningitis suggests that the molecule may play a role in some brain areas that regulate food intake and may mediate appetite reduction under inflammatory states. In this study, the CSF‐to‐serum ratio of LEAP2 in the patient with Listeria meningitis was markedly higher than that in the patient with bacteremia (0.532 versus. 0.081, respectively), suggesting the existence of a mechanism by which the LEAP2 level in CSF rises independently of that in serum.

Clinical guidelines recommend that when bacterial meningitis is suspected, empirical therapy should be commenced before CSF tests and CNS imaging are performed (Young and Thomas, 2018). Once antibiotics are administered, CSF cell counts or cell types may mimic those in aseptic meningitis, and cell culture results may become negative (Converse et al., 1973). Given that increased CSF LEAP2 levels are specific for bacterial meningitis, CSF LEAP2 measurement may be useful as an auxiliary diagnostic measure.

Limitations of this study include the lack of serum samples from patients with pneumococcal meningitis and the small number of CSF samples obtained during the course of routine clinical examination.

Considering LEAP2’s functions as an antimicrobial peptide and an antagonist of the ghrelin receptor, elevated CSF LEAP2 concentrations in bacterial meningitis suggest that LEAP2 may act against local bacterial infection or acute inflammation. Thus, increased LEAP2 levels during bacterial infection may be applicable as a potential biomarker. Furthermore, the presence of LEAP2 in the human CSF may suggest the existence of a peptidergic innate immune system in the CNS. This study should contribute to better understanding the host defense in bacterial meningitis.

## 5 CONCLUSIONS

CSF LEAP2 is increased in patients with bacterial meningitis, suggesting its biological significance in the pathology of CNS infections.

## 6 ETHICAL CONSIDERATIONS

We confirm that we have read the Journal's position on issues concerning ethical publication and affirm that this report is consistent with those guidelines.

## CONFLICTS OF INTEREST

The authors declare that they have no competing interests.

## AUTHOR CONTRIBUTION

Katsuya Sakai contributed to study concept and design, analysis and interpretation of data, drafting of the manuscript, and statistical analysis. Masamitsu Nakazato, Kazutaka Shiomi, Hitoshi Mochizuki, Md Nurul Islam, Hiroki Nabekura, Ryota Tanida, and Hideyuki Sakoda contributed to critical revision of the manuscript for important intellectual content.

### PEER REVIEW

The peer review history for this article is available at https://publons.com/publon/10.1002/brb3.2111.

## Data Availability

The data that support the findings of this study are available from the corresponding author upon reasonable request.
